# Intestinal Permeability in Inflammatory Bowel Disease: Pathogenesis, Clinical Evaluation, and Therapy of Leaky Gut

**DOI:** 10.1155/2015/628157

**Published:** 2015-10-25

**Authors:** Andrea Michielan, Renata D'Incà

**Affiliations:** Department of Surgical, Oncological and Gastroenterological Sciences, University of Padua, 35128 Padova, Italy

## Abstract

The pathogenesis of inflammatory bowel disease (IBD) is multifactorial with data suggesting the role of a disturbed interaction between the gut and the intestinal microbiota. A defective mucosal barrier may result in increased intestinal permeability which promotes the exposition to luminal content and triggers an immunological response that promotes intestinal inflammation. IBD patients display several defects in the many specialized components of mucosal barrier, from the mucus layer composition to the adhesion molecules that regulate paracellular permeability. These alterations may represent a primary dysfunction in Crohn's disease, but they may also perpetuate chronic mucosal inflammation in ulcerative colitis. In clinical practice, several studies have documented that changes in intestinal permeability can predict IBD course. Functional tests, such as the sugar absorption tests or the novel imaging technique using confocal laser endomicroscopy, allow an *in vivo* assessment of gut barrier integrity. Antitumor necrosis factor-*α* (TNF-*α*) therapy reduces mucosal inflammation and restores intestinal permeability in IBD patients. Butyrate, zinc, and some probiotics also ameliorate mucosal barrier dysfunction but their use is still limited and further studies are needed before considering permeability manipulation as a therapeutic target in IBD.

## 1. Introduction

The gut has a major role in food digestion and absorption as well as in maintaining the general homeostasis. It is estimated that the total bacterial cell count in our body exceeds ten times the total number of human cells, with more than one thousand species hosted in the gastrointestinal tract [1, 2]. The gastrointestinal microbiota, whose genome contains one hundredfold more genes than the entire human genome [3, 4], has important roles in nutrition, energy metabolism, host defense, and immune system development [5]. Indeed the altered microbiota has been linked not only to gastrointestinal diseases but also to the pathogenesis of systemic conditions such as obesity and metabolic syndrome [6, 7]. Therefore, the term “mucosal barrier” seems to properly highlight the pivotal role of the gut in the interaction with microbiota [8]: it is not a static shield but an active apparatus with specialized components. As stated by Bischoff et al. [9] “permeability” is defined as a functional feature of this barrier that on one hand allows the coexistence with bacterial symbionts necessary for our organism and on the other hand prevents luminal penetration of macromolecules and pathogens [10, 11]. Altered intestinal permeability has been documented during several conditions, namely, acute pancreatitis [12], multiple organ failure [13], major surgery [14, 15], and severe trauma [16], and could explain the high prevalence of Gram-negative sepsis and related mortality in critically ill patients [8]. Furthermore, perturbation of the complex mechanism of permeability has been associated with the development of irritable bowel syndrome [17–19] and steatohepatitis (NASH) [20, 21].

The pathogenesis of inflammatory bowel disease (IBD) is still unclear but in all probability is multifactorial and driven by an exaggerated immune response towards gut microbiome in a genetically susceptible host [22]. Increasing evidence suggests that intestinal permeability may be crucial [23, 24] and some authors even considered IBD as an impaired barrier disease [25].

## 2. Components of the Intestinal Barrier and Related Dysfunction in Inflammatory Bowel Disease

**Figure 1:**
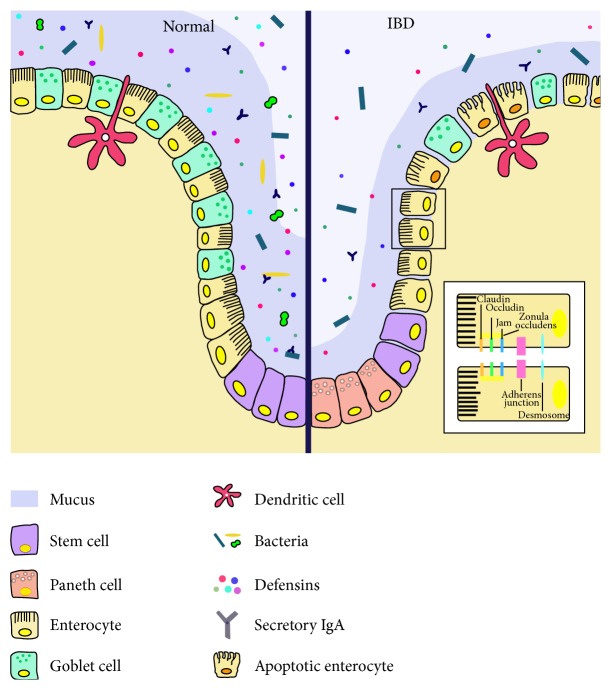
Components of the mucosal barrier in healthy gut (left) and inflammatory bowel disease (IBD) (right). For explanations see text. The basic structure of tight junctions and other junctional complexes are shown in the bottom-right box. JAM: junctional adhesion molecules.

The main component of the mucosal barrier is represented by the intestinal epithelium, which consists of a single layer of different specialized subtypes of cells [9, 22]: enterocytes, goblet cells, Paneth cells, and enteroendocrine cells but also immunity cells such as intraepithelial lymphocytes and dendritic cells (Figure 1). The mechanical cohesion of these cells and the regulation of paracellular permeability of ions and small molecules are ensured by three types of junctional complexes, namely, tight junctions (TJs), adherence junctions, and desmosomes [24, 26, 27].

IBD patients display increased paracellular permeability [28] with TJs abnormalities documented in several studies [29, 30]. These are complex multiproteins structures with an extracellular portion, a transmembrane domain and an intracellular connection with cytoskeleton (Figure 1). A decreased expression and redistribution of their constituents, including occludins, claudins, and junctional adhesion molecules (JAM), have all been documented in IBD [31–34] and a recent experimental mouse model found that deletion of claudin-7 initiates colonic inflammation [35]. Furthermore, tumour necrosis factor-*α* (TNF-*α*), one of the main effectors of IBD inflammation, may modulate the transcription of TJs proteins while its antagonists (anti-TNF-*α*) can ameliorate intestinal permeability [36, 37]. However, TNF-*α* leads to altered permeability also, inducing apoptosis of enterocytes, increasing their rate of shedding, and hindering the redistribution of TJs that should seal the gaps left [22, 38–41].

Goblet cells are specialized in the secretion of mucus which covers the surface of intestinal epithelium. Mucus is composed of proteins, carbohydrates, lipids, and a high degree of water but displays also antimicrobial properties thanks to antimicrobial peptides, mainly defensins produced by Paneth cells, and secretory IgA [24]. Ulcerative Colitis (UC) patients show a reduced number of goblet cells [42], a reduced thickness of the mucus layer [43, 44], and an altered mucus composition in terms of mucins, phosphatidylcholine, and glycosylation [45–48]. Moreover, altered Paneth cell distribution and function have been documented in IBD: these cells are normally restricted to the small intestines, within the crypts of Lieberkühn, but in IBD metaplastic Paneth cells may be detected in colonic mucosa, with subsequent secretion of defensins also in the large intestine [24, 49, 50]. However, the role of Paneth cells may be different in the two disease phenotypes since the expression of defensins is inducible by colonic inflammation in UC but is reduced in patients with colonic Crohn's disease (CD) [51]. Indeed, the diminished Paneth cell antimicrobial function might be a primary pathogenic factor in CD, particularly ileal CD [24, 43, 52, 53], while the increased secretion of defensins in UC may be a physiological response to mucosal damage.

## 3. Etiology of Permeability Dysfunction in Inflammatory Bowel Disease

Whether mucosal barrier impairment is a consequence of the inflammatory response or a primary defect that prompts mucosal inflammation is still under debate [54]. However, several studies suggest that altered intestinal permeability may be an early event in CD pathogenesis. First of all an augmented paracellular permeability has been found also in patients with quiescent IBD and correlated with intestinal symptoms even when endoscopic activity was absent [55]. Furthermore, an* ex vivo* study using Ussing chambers on colonic biopsies from CD patients [31] demonstrated a spatially uniform increase in transepithelial conductivity despite the presence of minimal mucosal erosions. This finding was attributed to the downregulation of TJs proteins. Finally, animal models of CD, namely, IL-10 knockout mice and SAMP1/YitFc mice, confirmed that increased permeability can be detected before the onset of mucosal inflammation [54].

On the other hand, genes involved in intestinal barrier homeostasis have been associated with IBD susceptibility [56] suggesting a genetic predisposition that is further supported by the observation that up to 40% of first-degree relatives of CD patients display an altered small intestinal permeability [57–62], with significant association with familial CD and NOD2/CARD15 variants [63, 64]. This gene, which is involved in bacterial recognition, modulates both innate and adaptive immune responses and is the main susceptibility locus for CD development [55]. Other studies have not found a correlation between permeability and genetic polymorphisms [60, 62, 65] but it is noteworthy that they have mostly involved sporadic CD cases. However, environmental factors too are main contributors in determining mucosal permeability since permeability is increased even in a proportion of CD spouses [61]. Moreover, a recent study highlighted the importance of age and smoking status rather than genotype in relatives [65]. Finally, to date there is only one reported case of CD development predicted by an abnormal permeability test in a healthy relative [66].

Independently from being genetically determined or caused by environmental factors, permeability impairment leads to the disruption of the physiological balance between mucosal barrier and luminal challenge [25] which cannot be adequately counteracted by innate immunity of IBD patients, which on the contrary responds with an aberrant immune activation [67]. As a matter of fact several defects in bacterial recognition and processing have been documented in CD patients carrying particular genetic polymorphisms, chiefly of pattern-recognition receptors such as NOD2/CARD15 [68, 69] and genes involved in autophagy like ATG16L1 and IRGM [70–72]. In intestinal mucosa, the lack of feedback between mutated NOD2/CARD15 expression and gut luminal microbiota can lead to the breakdown of tolerance [73]. Interestingly, a recent study by Nighot et al. demonstrated that autophagy is also involved in the regulation of TJs by degradation of a pore-forming claudin [74], linking autophagy to permeability.

Finally, intestinal microbiota* per se* is altered in IBD, particularly in its relative composition and diversity. This may represent a consequence of chronic mucosal inflammation but the influence of host genotype in shaping microbial community cannot be overlooked in CD [75] and NOD2/CARD15 genotype has been shown to influence the composition of gut microbiota in humans [76]. This dysbiosis may further aggravate permeability dysfunction by the loss of the symbiotic relationship between the microbiota and the mucosal barrier integrity [77].

## 4. Clinical Evaluation of Permeability in Inflammatory Bowel Disease

Permeability impairment may be early involved during the development of CD inflammation. Indeed, some of the known risk factors for disease relapse may induce inflammation through the increased mucosal permeability: this is a well-known mechanism for nonsteroids anti-inflammatory drugs (NSAIDs) [78] and recent evidence demonstrated that even stress acts in a similar manner through the release of corticotropin-releasing factors [79]. Furthermore, an impaired small intestinal permeability can predict the risk of CD relapse [80, 81] and patients with altered lactulose/mannitol test (L/M test) have 8-fold risk of relapse even if asymptomatic and with normal biochemical indices [82].

L/M test evaluates small intestinal permeability by measuring the urinary excretion after oral administration of these sugars. Lactulose is a large size oligosaccharide that usually does not have paracellular transport and can be adsorbed only in case of leakiness of intercellular junctions; mannitol is a smaller molecule that can freely cross the intestinal epithelium. Both the probes are equally affected by gastrointestinal dilution, motility, bacterial degradation, and renal function; thus, the ratio allows for correcting possible confounding factors [9, 82]. L/M test is used in clinical practice thanks to its noninvasiveness, its high sensitivity in detecting active IBD, and its ability to discriminate functional versus organic gastrointestinal disease [23, 83–87]. An altered L/M test has been reported in up to nearly 50% of CD patients [62]. Other sugars are routinely used to evaluate the upper intestinal tract, such as sucrose which is degraded by duodenal sucrase, thus reflecting the permeability of the stomach and the proximal duodenum [9, 88]. Therefore, multisugar tests have been developed, with the recent addition of sucralose, which is scarcely absorbed throughout the human intestine and thus allows a functional assessment of the whole intestinal tract, widening the possible application to UC [89].

**Figure 2:**
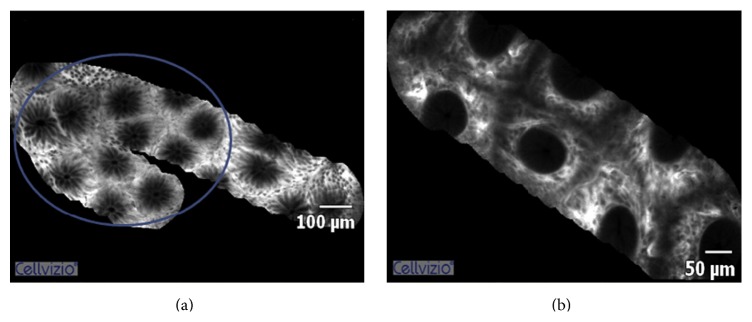
Confocal laser endomicroscopy images from a healthy subject (a) and an Ulcerative Colitis (UC) patient with inactive disease (b). UC patients display increased crypt diameter, intercryptic distance, and perivascular fluorescence. Courtesy of Dr. A. Buda and with permission of* Journal of Crohn's and Colitis* [105]; © inclusion under a Creative Commons license or any other open-access license allowing onward reuse is prohibited.

Other functional tests, such as 51Cr-EDTA [90–92] or the Ussing chambers [9], have demonstrated good accuracy but their invasiveness and the complex detection methods preclude their use in humans. Whereas promising results are shown by novel imaging techniques, particularly confocal laser endomicroscopy. this endoscopic technique allows an* in vivo* evaluation of the epithelial lining and vasculature with the use of intravenous fluorescein as a molecular contrast agent, which usually does not have paracellular transport [93]. Confocal laser endomicroscopy is now widely used in diagnosis and classification of gastrointestinal tumours [94–97] but it has also been applied in nonneoplastic conditions such as celiac disease [98], collagenous colitis [99], and irritable bowel syndrome [100]. The up to one thousandfold magnification permits detection of cellular and subcellular changes such as cell shedding [101], making it a powerful technique for the imaging of any defects in mucosal barrier in IBD. Confocal laser endomicroscopy demonstrated increased density of mucosal gaps after cell shedding in small bowel of CD patients [102] but also in macroscopically normal duodenum in both CD and UC [103]. Far from being just speculative findings these alterations may represent a subclinical impairment of intestinal permeability possibly predicting subsequent clinical relapse [104]. Recently confocal laser endomicroscopy has been applied in UC patients demonstrating that the occurrence of crypt architectural abnormalities can predict disease relapse in patients with apparent endoscopic remission (Figure 2) [105].

## 5. Permeability-Oriented Therapies

Agents routinely used in the therapeutic armamentarium of IBD may induce and maintain mucosal remission not only for their immunomodulating effect, but also through the restoration of epithelial integrity and permeability, as has been demonstrated for anti-TNF-*α* drugs in CD [37, 106]. Since similar effects have been obtained using elemental diets in CD [107, 108], increasing interest relies on dietary approaches with the use of immunomodulatory nutrients and probiotics.

Western diet with its high content of fat and refined sugars is a risk factor for the development of CD [109] probably inducing a low-grade inflammation via gut dysbiosis and increased intestinal permeability [110–112]. Furthermore, there is increasing concern about the role of industrial food additives as promoters of immune-related diseases. A recent review showed the ability of additives to increase intestinal permeability by interfering with the TJs, promoting the passage of immunogenic antigens [113]. On the contrary, certain fatty acids (propionate, acetate, butyrate, omega-3, and conjugated linoleic acid), amino acids (glutamine, arginine, tryptophan, and citrulline), and oligoelements, essential for intestinal surface integrity, when supplemented to experimental models of gut diseases, can reduce inflammation and restore mucosal permeability [114]. However, their therapeutic efficacy, particularly in IBD, remains debatable: butyrate, zinc, and probiotics have the strongest evidence in this regard.

Butyrate is a short chain fatty acid produced by intestinal microbial fermentation of dietary fibres [115] which in experimental models stimulates mucus production and expression of TJs* in vitro* but a wider range of action is expected [116–120]. It is so crucial for general homeostasis of enterocytes that its deficiency, measured as faecal concentrations, has been taken as an indirect indicator of altered barrier function [9, 121, 122]. In clinical practice topical butyrate had proved efficacy in refractory distal UC [123].

Other fatty acids with similar properties have also been proposed as an adjuvant therapy in IBD, namely, omega-3 and phosphatidylcholine [124–126], but their use in clinical practice is still limited.

Zinc is a trace element essential for cell turnover and repair systems. Inflammatory conditions and malnutrition are known risk factors for zinc deficiency and several works proved the efficacy of its supplementation during acute diarrhoea and experimental colitis [127–129]. We have shown that oral zinc therapy can restore intestinal permeability in CD patients probably through its ability to modulate TJs both in the small and the large bowels [130, 131].

The rationale for the use of probiotics in IBD is the aforementioned dysbiosis that characterizes these diseases. Several trials have tested the efficacy of different species of probiotics in IBD, with conflicting results. To date the ones with proven efficacy are* Escherichia coli Nissle 1917*,* Bifidobacterium*,* Lactobacillus rhamnosus GG*, or the multispecies VSL#3 which contains eight different probiotics [132]. Yet their use is still limited to UC and often aimed at maintaining remission rather than treating active disease, as highlighted by the meta analysis by Jonkers et al. [133]. The mechanisms of their effect in UC have not been fully understood but probably, along with direct anti-inflammatory effects, they may strengthen mucosal barrier [134, 135] and reduce intestinal permeability once again upregulating TJs proteins [136, 137]. Even the beneficial effect of probiotics in pouchitis seems to be related to the enhancement of mucosal barrier function [138]. Another potential mechanism of action is the restoration of butyrate-producing bacteria: UC patients have reduced bacterial species like* Faecalibacterium prausnitzii* [133, 139] and supplementation with butyrate-producing species or probiotics along with preformed butyrate showed efficacy in experimental models [140, 141].

Finally, vitamin D is worth a mention because it is involved in maintaining intestinal barrier function [113] and polymorphisms of its receptor have been associated with the development of IBD [142, 143]. While the expression of vitamin D receptor on intestinal epithelium inhibits inflammation-induced apoptosis [144], its deletion leads to defective autophagy that promotes experimental colitis [145]. However, further data and clinical trials are needed to rationalize vitamin D use in IBD management [146].

## 6. Conclusions

The impairment of intestinal barrier function is one of the key events in the pathogenesis of IBD. Whether it precedes and predisposes disease development is still under investigation, especially in CD, but surely it perpetuates and enhances chronic mucosal inflammation by increasing paracellular transport of luminal pathogens.

Novel functional and imaging techniques allow us to assess mucosal permeability* in vivo* and help identifying patients at risk of relapse guiding therapeutic management.

Manipulation of intestinal permeability is an intriguing therapeutic approach but more studies on efficacy and safety are warranted before nutritional immune-modulators can be used in clinical practice.
